# Implementing a quality improvement programme in palliative care in care homes: a qualitative study

**DOI:** 10.1186/1471-2318-11-31

**Published:** 2011-06-09

**Authors:** Sue Hall, Cassie Goddard, Frances Stewart, Irene J Higginson

**Affiliations:** 1 King's College London, Department of Palliative Care, Policy & Rehabilitation, Cicely Saunders Institute, Bessemer Road, Denmark Hill, London, UK

## Abstract

**Background:**

An increasing number of older people reach the end of life in care homes. The aim of this study is to explore the perceived benefits of, and barriers to, implementation of the Gold Standards Framework for Care Homes (GSFCH), a quality improvement programme in palliative care.

**Methods:**

Nine care homes involved in the GSFCH took part. We conducted semi-structured interviews with nine care home managers, eight nurses, nine care assistants, eleven residents and seven of their family members. We used the Framework approach to qualitative analysis. The analysis was deductive based on the key tasks of the GSFCH, the 7Cs: communication, coordination, control of symptoms, continuity, continued learning, carer support, and care of the dying. This enabled us to consider benefits of, and barriers to, individual components of the programme, as well as of the programme as a whole.

**Results:**

Perceived benefits of the GSFCH included: improved symptom control and team communication; finding helpful external support and expertise; increasing staff confidence; fostering residents' choice; and boosting the reputation of the home. Perceived barriers included: increased paperwork; lack of knowledge and understanding of end of life care; costs; and gaining the cooperation of GPs. Many of the tools and tasks in the GSFCH focus on improving communication. Participants described effective communication within the homes, and with external providers such as general practitioners and specialists in palliative care. However, many had experienced problems with general practitioners. Although staff described the benefits of supportive care registers, coding predicted stage of illness and advance care planning, which included improved communication, some felt the need for more experience of using these, and there were concerns about discussing death.

**Conclusions:**

Most of the barriers described by participants are relevant to other interventions to improve end of life care in care homes. There is a need to investigate the impact of quality improvement programmes in care homes, such as the GSFCH, on a wider range of outcomes for residents and their families, and to monitor the sustainability of any resulting improvements. It is also important to explore the impact of the different components of these complex interventions.

## Background

Populations across the world are ageing. The very oldest people often experience multiple chronic diseases, such as heart failure, respiratory failure and dementia. As a result of physical and mental frailty, in economically developed countries, older people are increasingly cared for in nursing or residential homes at the end of life. The majority of residents in nursing homes die within 2 years [1]. They die with multiple medical pathologies, but not always from them (as evidenced for people with dementia, who in nursing homes account for 70% of the population). Literature reviews have shown a considerable need for palliative care in these settings, including the need for improved symptom management[2-4] and addressing psychosocial and spiritual needs [3,5]. There are, however, barriers to providing palliative care in care homes, for example: staff shortages and turnover; lack of time and knowledge of palliative care among staff; shortage of equipment; lack of support from primary care; and poor communication among staff, and between staff and residents and their families [6-8]. Some of these are likely to be encountered across different countries, however, since the systems of care and support for residents are different (e.g. residents' access to medical and palliative care support), others may be more context specific.

Care homes in England offer nursing care and/or personal care to older people. They are owned and managed by a range of public sector, private sector, and not-for-profit bodies, and are registered with, and regulated by, the Care Quality Commission. The proportion of deaths in care homes increases with age [9]. In England (2006-2008) 10.5% of people aged between 75-79 years died in care homes, whereas 36.9% of those aged over 90 years died in these settings. Delivering good end of life care in care homes depends on a range of structural, political, cultural and resource issues [10]. A study to map the wider health and social system surrounding care homes showed that the quality of the interrelationships with this wider system determines the quality of the end of life care they can provide. For example, leadership was important, and knowledge of and access to outside resources and expertise was variable.

Over the last 15 years there have been a range of developments to improve end of life care in care homes. These include providing education and training for care home staff [11], quality initiatives [12], and giving clinical nurse specialists a higher profile [11,13]. One recent approach developed in the UK to improve end of life care in care homes is the Gold Standards Framework (GSF). This is a multidimensional quality improvement programme developed to optimise end of life care in generalist settings as part of the UK End of Life Care Strategy [14]. The programme has spread internationally with pilots in Australia, New Zealand, USA, Canada, Belgium, Holland, and has been adapted to address the needs of residents in care homes for older people (GSFCH). Further details of the GSFCH are in Table 1. The GSFCH uses similar key tasks (the 7C's, shown in Table 2), templates and assessment tools as the GSF, with some modifications to make them more suitable for a care home environment. The programme has developed over six phases http://www.goldstandardsframework.org.uk/GSFCareHomes, and has developed a quality improvement process leading to accreditation. A survey conducted as part of the evaluation of the first large scale implementation (Phase II) showed a reduction in hospital deaths reported by the 46% of homes providing the necessary data [15,16], and improvements on other indicators, such as having an up to date register for end of life care [17]. By June 2009, nearly 1,000 homes had undertaken training across the country, with up to 100 being accredited each year. Evaluations of the implementation of the GSFCH in Scotland [18,19] and in Manchester [20] have reported positive findings. For example, the introduction of the GSFCH in Scotland resulted in a reduction in hospital deaths and increased 'do not attempt resuscitation' documentation and advance care planning. The evaluation of the implementation of the GSFCH and the Liverpool Care Pathway (LCP) for people with dementia in Manchester describes an emerging picture of overall staff confidence in the planning and implementation of end of life care and satisfaction with education and training and GSF/LCP tools.

**Table 1:** The Gold Standards Framework for Care Homes

The GSFCH is a multidimensional framework of enabling tools, tasks and resources used in care homes for older people, with training and central support from the GSF team and local support from a GSFCH facilitator.
Rather than being prescriptive, the GSFCH can be adapted to meet local needs. The focus is on organising and improving the quality of care for care home residents in the last year of life in collaboration with GPs, primary care and specialist palliative care teams.
Key elements include multidisciplinary resident review meetings, completion of a prognostic register (to prompt discussion of life expectancy and care planning) and associated advance care planning, and discussion of resuscitation status.
The target outcomes are improvements in advance care planning, communication team working, reducing the number of residents being transferred to hospital, and high quality clinical care.

**Table 2:** The 7Cs

C1	Communication	Identify patients in need of palliative care, regular meeting to discuss with team and/or primary health care trust, label notes, use advanced care plan
C2	Co-ordination	Allocated co-ordinator for whole, key carer/link person/link worker for each patient
C3	Control symptoms	Use of assessment tool, agreed note keeping and action points, equipment standard and PRN (taken only as needed) medication
C4	Continuity	Form sent to out of hours provider, patient held record or medication card
C5	Continued learning	Regular review/audit of last deaths using significant event analysis, programme of ongoing training, library resource
C6	Carer support	Staff issues and learning points + feedback after death, all staff supported
C7	Care in the dying phase	Modified Liverpool Care Pathway implemented in last days of life, agreed practice for notification of relatives, death certification and after death care, support for the bereaved families, support for staff and other residents as needed

However, nearly half of the care homes in the Phase II evaluation may not have completed implementation of the GSFCH, or may have withdrawn from the programme [17], suggesting that they encountered barriers to implementation. Relatively little is known of these. The aim of this study is to explore the views of care home staff, residents and their families on the benefits of and barriers to implementation of the GSFCH, to inform the development of palliative care interventions in care homes for older people.

## Methods

Since, relatively little is known of the perceived benefits and barriers to implementing the GSFCH and other palliative care programmes in care homes, we used qualitative methods to explore participants' views in-depth. We chose to use individual interviews rather than focus groups because we felt that focus groups would be impractical. We felt that staff would be more reluctant to voice individual concerns in a group from the same care home, and that it would be very difficult to arrange groups involving several care homes. From our previous experience of interviewing residents, we knew that it was difficult to find a time when they are willing and able to take part in research.

### Ethical approval

The study was approved by the King's College Hospital Research Ethics Committee (REC Refs: 07/H0808/136 & 07/Q0703/89) and met the requirements of the Local Research Governance Framework. To protect the anonymity of participants we used our identification number for the homes and whether they were in earlier or later phases of the GSFCH, rather than participant names when using quotes.

### Setting

All care homes for older people in two London boroughs were invited to take part in this study. Both boroughs were rated on at least one summary measure of deprivation as being in the top 50 deprived areas in England. One was rated within the top 10 social deprivation areas. Homes were identified through the website of the regulatory body for care homes in England and the local Care Home Support Team. Care home managers were sent information about the study, then contacted by telephone to ask if they were willing for their home to be included. All nine care homes which were, or had been, involved in the GSFCH took part. Their participation in the GSFCH programme was verified by the GSFCH team. All homes had on-site nursing and were privately funded (not funded by a charity or publicly funded by the National Health Service). Eight homes worked with a single general practice, one home worked with two practices. Further details of the homes are given in Table 3. At the time of the study, one of these homes had withdrawn from the GSFCH Programme (at an early stage), and another had temporarily withdrawn from the GSFCH programme. Support from the local hospice and the Care Home Support Team was available to all the care homes.

**Table 3:** Characteristics of care homes

GSFCH Phase	Size (Beds)	Approximate time in GSFCH (months)	Participated in the study					
			Manager	Nurse	Care Assistant	Resident	Family	TOTAL
II	49	26	1	1	1	0	0	3
II	62	Withdrawn from programme	1	1	1	2	1	6
III	55	15	1	1	1	2	1	6
III	39	17	1	1	1	2	2	7
IV	92	3	1	1	1	2	0	5
IV	93	5	1	1	1	0	0	3
IV	58	10	1	0	1	0	0	2
IV	88	5 (temporarily withdrawn)	1	1	1	3	3	9
IV	128	9	1	1	1	0	0	3
TOTAL			9	8	9	11	7	44

### Participants

In total, 44 people were interviewed - 9 care home managers, 8 nurses employed by the homes, 9 care assistants, 11 residents, and 7 residents' family members.

We interviewed the manager and a care assistant from each home, and a nurse employed in each of the eight homes. We were unable to recruit a nurse in one home (although this home had on-site nursing). Care assistants and nurses were randomly selected from staff lists. In addition 11 residents (two from a Phase II, four from Phase III, and five from Phase IV homes) and seven residents' family members (one from a Phase II, 3 from Phase III and 3 from Phase IV homes) were interviewed. Six family members represented residents who were too ill or cognitively impaired to take part. The number of participants from each home is shown in Table 3.

### Interviews

Semi-structured interviews were conducted by CG or FS, both of whom had experience of conducting qualitative interviews and were trained by SH to use the topic guide for this study. Staff and residents were interviewed in the care home, family members were interviewed either in the care home or in their own homes. To cover the main aspects of the GSFCH, the topic guide was based on the seven key tasks of the GSFCH: the 7Cs (Table 2). In addition to broad questions about the GSFCH in general and the 7Cs, specific questions were asked about some GSFCH tasks. Examples of questions and prompts from the topic guide are given in Additional File 1. These reflected key standards in the GSFCH accreditation checklist [21]. Although the topics covered were similar for the care home staff and residents and their families, topic guides were adapted for each group. They were a flexible framework of questions which allowed the interviewers to adapt the questions to the interest, knowledge and language ability of participants. The average time taken to conduct the interviews was: 57 minutes for care home managers; 53 minutes for nurses; 43 minutes for family members; 40 minutes for residents; and 31 minutes for care assistants. Participants were given a £20 gift voucher to compensate them for their time. Interviews were recorded and transcribed verbatim.

### Analysis

The Framework approach to qualitative analysis [22] was used. Further information on this approach, our treatment of the interview transcripts, and the development of the thematic framework is given in Additional File 2. The analysis was deductive: all themes were developed *a priori*, based on the topics covered in the interview (7Cs). This was to enable us to consider benefits and barriers of individual components of the programme, as well as of the programme as a whole. This approach follows the recommendations by the MRC framework for the development and evaluation of complex interventions, in trying to determine the mechanisms of action of different interventions [23]. In qualitative analysis there is considerable diversity in the identification of themes, the interpretation of the concept, and its function in data analysis. This is partly due to the different theoretical approaches to qualitative analysis. We defined "theme" as qualitative data grouped around a central issue - a more limited and concrete category than is often used in qualitative research. All transcripts were indexed (coded) by FS using the final agreed framework. Indexing was checked by SH and CG, and any disagreements were resolved by discussion. Charts containing extracts of interviews were constructed and used to describe the range of participants views in each theme and sub-theme. The findings were linked back to the literature.

## Results

### Response rates

Response rates were 9/9 for managers, 9/15 for care assistants, 8/15 for nurses, 11/28 for residents and 7/25 for family members. The denominator is the number of people invited to take part. We did not ask individuals to justify their decision not to take part, however, care home staff often said that they were too busy to take part.

### Qualitative analysis

The main themes are summarised in Table 4 and quotes from participants to illustrate these are in Table 5. These were selected to illustrate the diversity in each theme.

**Table 4:** Main themes

General views on the GSFCH	
Communication	Communication with general practitioners
	Communication with others
	Supportive care register
	Coding predicted stage of illness
	Team meetings
	Advanced care planning
Co-ordination	Role of the palliative care lead
Control symptoms	Assessment and management
Continuity	Out of hours handover form
Continued learning	Training and significant event analysis
Carer support	Staff and residents' bereaved family
Care in the dying phase	Liverpool Care Pathway for the last days of life

(Benefits and barriers to each of these were indexed separately)

**Table 5:** Quotes from participants

General views on the GSFCH	
Q1	"If you're running a competitive business ... you've got to develop something that's special ... it has helped the home acquire more clients ... in the past there were difficult times, so it needed that to actually boost it - the reputation a bit, you know, and restore the confidence of people... (Manager of Earlier Phase home 13)
Q2	... I am still in the early stages really, it's a slow process ... I think it would be very difficult, because if you don't have an understanding of palliative care, the terminology that they use on all the paperwork, there's a lot of paperwork, I think I would have struggled ...We've been fortunate that there is a facilitator in the care home support team ... but I feel for it to be effective, there should be more input. I think with the managers, it's time management ... lots of managers would like to do it, but the time it takes to discuss with staff and get all the paperwork, discussion, discussion, discussion, and unfortunately, that is time, and we don't have that time. (Manager of Earlier Phase home 03)
**C1: Communication**	
Communication with GPs	
Q3	I think our GP has done a fantastic job ... he's given us his printed history of the medical episodes of each patient ... all the medical histories, all the drug changes ... (Manager of Later Phase home 21)
Q4	He is a young man. He is very nice, and he listens to what you want to say. He doesn't wash you off as any old thing. He is a really lovely man. First class. You couldn't have a better doctor in the world. He is wonderful. (Resident in Earlier Phase home 28)
Q5	The GP was a problem ... because Gold Standard Framework, in order for it to be running well, you need a GP that's cooperating. So the GP needs to take it as their responsibility. Because we needed to involve them - that was the difficulty, you know, because of their time. There was the lazy response and things like that ... And also the cooperation of the family as well, that's- that's monumental because obviously some families, as you know, will not visit. (Manager of Earlier Phase home 13)
Q6	... and I must tell you straight, he's not my idea of a doctor. Doesn't show any interest in what's going on at all ... I should say four minutes was all the time the man was in the room and away he went. (Resident in Later Phase home 31)
Q7	[name of doctor] came here to see her [resident], he was assessing her ... but he hasn't got back to me ... I don't think ... what his results are, and this is very frustrating. (Daughter of resident with dementia in Later Phase home 31)
Communication with others	
Q8	Some of the carers especially, they seem to be afraid sometimes of reporting things or saying things in case it's the wrong thing, that they'll be blamed, and that's something we're trying to get over ... to them because, at the end of the day, they spend more time with the residents than anybody else ... (Manager of Earlier Phase home 30)
Q9	...there's one of them can't even speak English and I can't get through to her, the one nurse, you know ... (Resident in Earlier Phase home 13)
Supportive care register	
Q10	It works well ... from that point [going on to the supportive care register] there's no need to ask questions or whatever. We know what we're doing because we communicate with each other). (Care Assistant working Earlier Phase home 30)
Q11	... I just feel it needs to be more explicit [supportive care register] ... but that depends on the nurse - but I just also feel that their needs to be ... a guideline to say what to record ... (Manager of Earlier Phase home 13)
Coding predicted stage of illness	
Q12	There is a separate folder ... which you can go and look at to see how the residents are colour-coded. I find this very useful. If you know someone is dying, you care for them sensitively and are aware of their needs and also their families' needs. (Care assistant working in Earlier Phase home 03)
Q13	... we struggled at first [with coding stage if illness] because nobody wants to think in those terms, but I think ... it's something that's coming a bit more naturally now. (Manager of Later Phase home 33)
Q14	My mum's 84 now, she could last another 10 years couldn't she? (Daughter of resident in Later Phase home 31)
Q15	This lady that died, they only gave her a couple of months, she was here two and a half, two years with us. (Nurse working in Earlier Phase home 03)
Team meetings	
Q16	Now, I think we have a problem with that. Um, and that's because of the meetings not being as regular as they should be. And that's why we're sort of picking up and dealing with that now, so we can sort of get up and back on track ... I think it should be fine, and get - as you say, pin the GP down (*laughs*) and get him into a regular routine of the meeting. (Manager of Earlier Phase home 03)
Q17	I don't think there was any sort of coordination involving the families anyway. What went on behind the scenes I don't know ... would like to have been part, more part of it ... there should be, I think a periodic meeting between at least one representative of the family and somebody representing the care home and maybe, maybe the doctor or whatever ... (Son whose father had recently died in Earlier Phase home 30)
Advance care planning	
Q18	... so we can really establish, otherwise ... no questions, why did you leave them in the home? Why we didn't, why did they send to the hospital, but if it's there, and it really made their wish then we are covered. (Nurse working in Later Phase home 21)
Q19	It's upsetting and depressing even talking about it. (Niece of resident living in Earlier Phase home 30)
Q20	For me its difficult to accept but if residents are willing to discuss [advance care plans], then by all means ... But not all individuals will want to discuss it. (Care assistant working in Early Phase home 03)
Q21	Some of the carers might not be, because of culture or whatever, might not be in a (pause) I want to ... say it kindly ... a good frame of mind to accept death, because... they look on it in a different, a completely different way in their culture ... cos most people do and some wouldn't not like to talk openly about death. They see that as a taboo subject, so that's one of the barriers as well ... actually using the word 'death' and 'dying' that's an obstruction as well, a lot of people don't use those two words. (Manager of Earlier Phase home 13)
Q22	I don't want no-one to come and talk with me about that, I just don't, that's ... cos it'll start me off on getting me depressed. (Resident in Earlier Phase home 13)
Q23	It [advance care planning] might seem like a good idea to me. But again, it's down to the implementation. I think ... just tokenism. (Son who's father had recently died in Earlier Phase home 30)
Q24	... all I have to send to the hospital with them is this piece of paper and I think half the time the doctors and the nurses don't even look at this in hospital because like the gentleman I was telling you about that we sent to hospital - had they looked on this piece of paper, that he has written down there that he didn't want to be resuscitated they would've like not gone through so much intervention. (Manager of Earlier Phase home 13)
**C2: Coordination**	
Q25	Well, it works well ... he [GSFCH lead] comes back and shares that information with us, and you know, what we have learnt, we have different links for each floor as well, so they link into him. (Manager of Earlier Phase home 13)
Q26	... amongst everything else I've got to do ... I think somebody else should be doing it. (Manager of Earlier Phase home 30)
**C3: Control of symptoms**	
Q27	...assessment will get better if we use a tool designed specifically for end of life. (Manager of Later Phase home 21)
Q28	... our patients have some dementia ... you already have a difficulty addressing symptoms in mass you know, because they can't verbalise "I've got pain," and some of them can't verbalise they've got pain so you have to rely on non-verbal signs to identify pain and with a very confused patient, that could be very, very difficult. You know, so ... we - I think we do have to be very careful of how we are assessing pain, you know, and hopefully make a tool work for that resident. (Manager of Later Phase home 21)
Q29	... varies sometimes from nurses to nurses as well. The issues is that sometimes it's difficult to tell, you know, they psychol - you know, but certainly when it comes to assessing those things, you know, the nurses would carry on that, you know. But they're, depending on the way a different individual interprets that...sometimes can be different, you know. (Manager of Earlier Phase home 13)
Q30	[nurse from hospice] comes in to give us a bit of support, and whenever we have a client that comes in and we feel that, you know, the pain area must ... we always contact them [the hospice] and then we get their advice and everything, and we move forward in liaison as well with the hospital and the GP. (Manager of Later Phase home 34)
**C4: Continuity**	
Q31	... it gives guidelines to the out of hours GP when he comes to know what the way forward is. Do I attend for this resident and send to hospital? Is it because maybe there's a chest infection that can be treated and they can come back, or is it you know, the culmination of the disease condition - that the person is progressing that way, and there's nothing much that the hospital can do? It helps him make a decision of whether to send a client to hospital or not. (Manager of Later Phase home 34)
**C5: Continued learning**	
Q32	... you are learning to become aware, so that if you have somebody who is a culture which you are not familiar with at least you know how to cope with it, and when they are performing whatever rites they have to do you don't, you don't look down on them or you don't just, it's because some people might think, oh I'm a Christian I don't have to do this, but it's their right ... (Nurse in Later Phase home 31)
Q33	They [care assistants] ought to have a proper training period ... so they've got a certificate to say they've been through it... and also regular training as well - refreshers, like teachers have to do ... Now there, there is a problem there with money ... because who's going to pay for all this? (Son whose father had recently died in Earlier Phase home 30)
**C6: Carer support**	
Q34	I suppose sometimes with the workload it's a bit difficult and sometimes, you know, they come on duty and they've got family commitments and then sometimes you want to sit down with them and talk to them, and at the same time there's work to be done. (Manager of Later Phase home 34)
Q35	I don't think that's anything to do with them [emotional support from staff], some people might do, I don't know ... as I say I'm a more down to earth pragmatic sort of person, I like to get on with it myself. But other people might need it, because they knew their parents I suppose. But again, is it their business? I'd rather they spent their energies on looking after the old folk who are still alive than worrying about me. (Daughter of a resident with dementia in Later Phase home 31)
**C7: Care in the dying phase**	
Q36	I think it's something very good, if it is explained, and people understand. ... I find it most, however, promoting good communication ... Promoting thinking and better planning, including psychological, social and spiritual ... (Nurse working in Earlier Phase home 30)
Q37	...the only time I can implement it [LCP] is when somebody is going to die, I thank...that it doesn't happen quite often here. So when it gaps like that you need to retrain the memory. (Manager of Later Phase home 21)

### General views on the GSFCH

Staff in all the Earlier Phase (II & III) homes felt there were benefits to the programme. These included: improved symptom control for residents; better team communication; finding helpful external support and expertise; increasing staff confidence; fostering choice for residents (reducing unnecessary hospital admissions and allowing residents to die in the home); and boosting the reputation of the home (Quote (Q)1). Staff in both the homes which had withdrawn from the programme were keen to re-join. Barriers to implementing the programme reported by staff included: the amount of paperwork; lack of understanding of the palliative care terminology used in the GSFCH paperwork (Q2); time and money; and staff believing that palliative care was only for residents with cancer. The manager of one of the Earlier Phase homes felt that progressing through the programme was a slow process and that having previous experience in palliative care was important to success (Q2). Although Later Phase homes had less experience, and therefore less to say about their experiences in the programme, they were optimistic that it would be beneficial.

### C1: Communication

#### Communication with general practitioners

For care homes to deliver good end of life care there needs to be effective communication and collaboration. Many of the tools and tasks in the GSFCH focus on improving communication. Developing such relationships with general practitioners (GPs) is a key component of the GSFCH. Although some homes had good collaborative relationships with GPs (Q3), and one of residents was delighted with the communication with their GP (Q4), six managers described problems trying to achieve this (Q5). Residents and their families also sometimes described problems with GPs. For example, a resident of the Later Phase homes felt that their GP showed little interest in the problems she was having with the circulation in her feet and legs and did not spend enough time with her in the consultation (Q6). The daughter of a resident with advanced dementia in the same home was concerned that she had not been given information about her mother's condition (Q7). We do not know if this was the same GP.

#### Communication with others

Staff described communication and collaboration with a wide range of external collaborators, including: palliative care specialists from local hospices and hospitals; district nurses; the care home support team; physiotherapists; mental health specialists; counsellors; and complimentary therapists. They also describe internal collaborative relationships between their own link nurses and between managers and other care home staff. Barriers to communication and collaboration included: workload; staff shortages; and care assistants not feeling confident to report problems due to concerns about being blamed (Q8). For many care home staff, English was not their first language, therefore poor English was another barrier to communication (Q9).

#### Supportive care register

A supportive care register is a summary document which can help facilitate communication between everyone involved in a resident's care. For each resident on the register there is, for example, information on prognosis (which is coded); any problems or concerns; anticipated needs; and residents' preferred place of care. It is a central place to keep all facts to hand for team discussion, and acts as a prompt in proactive planning. Staff agreed that having a supportive care register helped to improve communication. It provided: an opportunity to discuss end of life issues with residents; facilitated interdisciplinary teamwork; and helped them monitor residents' progress more efficiently (Q10). However, three participants felt they needed more experience or guidance to complete the relevant forms. Two of these were in Earlier Phase homes (Q11). Other barriers were people not feeling comfortable talking about dying (see Advance Care Planning) and difficulty in involving families (see Q5).

#### Coding the predicted stage of illness

Having a coding process to identify the estimated predicted stage of illness and likely needs at each stage is another tool with the potential to facilitate communication. The main perceived benefits of this were: increasing staff awareness of residents who are dying; their current and future needs and those of their families (Q12). However, some staff had felt uncomfortable about trying to identify residents nearing the end of life (Q13), or felt that accurate estimation of predicted stage of illness was difficult (Q14). To illustrate this point, several people described cases where residents had "bounced back" and lived much longer than expected (Q15).

#### Team meetings

Regular interdisciplinary meetings provide an opportunity to discuss residents on the supportive care register and to plan care. No benefits to such meetings were described. Staff described some problems in organising meetings, particularly if they wished GPs to be involved (Q16). The son of a resident who had recently died felt he would like to have attended such meetings and been more involved in discussions about his father's end of life care (Q17).

#### Advance care planning

Communication with residents and their families can involve difficult advance care planning discussions. Staff perceptions of the benefits of advanced care planning included: increasing family involvement; helping prevent confusion when residents are rushed to hospital (particularly out of hours); and protecting the home from blame for not sending a resident to hospital (Q18). Perceived barriers included: difficulty in deciding when residents have the capacity to make such decisions; disagreements within families; night staff ignoring plans and sending residents to hospital regardless of their preferences; and residents and their families and care home staff being unwilling to discuss death (Q19, Q20). Since care home staff are usually ethnically diverse, it is important to consider cultural differences in willingness to discuss death (Q21). We interviewed two residents and a family member in one of the Earlier Phase homes using GSFCH advance care planning documentation, and none recalled completing such a document. However, both the residents felt uncomfortable about talking about death and neither wanted to complete a plan (Q22). The son of a resident who had died recently in an Earlier Phase home felt that advanced care planning was a good idea, but questioned whether this would make any real difference (Q23). There was some support for his concern from a manager, who describes an incident where a residents wish to not be resuscitated was ignored in the hospital (Q24).

### C2: Coordination: the palliative care lead

Having a palliative care lead or coordinator can act as a link in the care home and with various external professionals. In GSFCH this person acts as a link between the GSFCH facilitator and the rest of the care home staff, and organises meetings etc. Benefits of and barriers to having a lead were described by Earlier and Later Phase homes. Benefits included: more effective communication and organisation of end of life care; timely access to advice; disseminating knowledge from GSFCH workshops to staff (Q25); and making sure that staff attend team meetings. The main barrier was lack of time to take on this role (Q26).

### C3: Control of symptoms: assessment and management

Assessing and managing symptoms is a key component of end of life care. The GSFCH recommends the use of a range of assessment tools to assess physical and psychological symptoms, including a tool specifically for residents with dementia. Staff felt that using such tools improved communication and planning, and that having a symptom assessment tool developed specifically for end of life would be particularly useful (Q27). The main perceived barriers to using them were difficulties in assessing symptoms for residents with dementia (Q28) and variability in interpretation of the measures (Q29). This manager refers to psychological symptoms, although staff usually talked of pain when they discussed the management of symptoms. Care homes often depended on external expertise for symptom management, usually this was from the GP or the local hospice. The latter they found particularly helpful (Q30).

### C4: Continuity: the out-of-hours handover form

Good end of life care requires continuity of care. Residents often need to see a GP out-of-hours, and documents such as an out-of-hours handover form can be a valuable tool to facilitate continuity of care. Such forms are usually completed by a multidisciplinary team and sent to GP practices or the out-of-hours providers, and sometimes others such as the ambulance service. Staff did not raise any major problems with using these forms and felt that they succeeded in preventing residents being sent to hospital inappropriately (Q31).

### C5: Continued learning: training and significant event analysis

The GSFCH has a well developed curriculum which includes resources, learning aids and tools with adaptations to meet the needs of local areas. Most staff described some training in end of life care. The extent and type of training varied considerably between homes. The homes in this project are situated in areas of high ethnic diversity, and the nurse in one of the Later Phase homes found training in cultural differences in end of life care particularly helpful (Q32). Culture and language were also seen as barriers to staff education, along with not having the time or resources to attend training. It is also important that plans for staff education include care assistants since they provide much of the day-to-day care of residents (Q33). Reflective learning, for example, using significant event analysis techniques can occur at notable points in the care of residents. These are intended to be non-judgemental group exercises, however, some members of staff might be unwilling to voice their concerns when they feel that a resident's care has not been optimal (see Q8).

### C6: Carer support: staff and residents' bereaved family

End of life care does not end when a resident dies; bereavement care for residents' families and friends, care home staff and other residents is important. One Earlier Phase home had a full time counsellor who provided bereavement care for residents' families and friends, care home staff and other residents. This home also provided written information developed for the GSFCH. Other homes described more informal chats with staff, tea and sympathy, and attending funerals rather than specific bereavement plans. One of these had provided bereavement counselling for staff. The main perceived barriers to providing bereavement care were: reluctance to talk about death; and lack of time to provide such support (Q34). The daughter of one of the residents with dementia was also aware of the time constraints on staff and felt that caring for residents should take priority over caring for their families (Q35).

### C7: Care of the dying: the Liverpool Care Pathway

An increasing number of care homes are using integrated care pathways such as the Liverpool Care Pathway for the final days of life. We asked staff specifically about the Liverpool Care Pathway. Staff were generally positive about the pathway and felt that it promoted good communication and helped residents' families (Q36). The main perceived barriers to using it were: difficulty in predicting when residents were reaching the last days of life; staff being reluctant to start using new forms of documentation; and becoming de-skilled when deaths in their care home were relatively infrequent (Q37).

## Discussion

Although staff working in these homes perceived a range of barriers to implementation, they were generally positive about the GSFCH and were optimistic that the tools and strategies they were using, or planning to use, would result in better end of life care for residents. They spoke of improved communication, increased staff confidence in end of life care, increased access to valued specialist palliative care support, fostering choice for residents and increased awareness of residents who were dying and their needs. Improved communication, increases in staff confidence and more positive attitudes to end of life care have been noted in evaluations of the GSFCH [16,18,20,24]. Staff in most homes seemed to want to provide the best possible care for their residents and to be seen to do so. Being able to say that they had reached the "gold standard" could also be seen as good for business, thus being accredited could be highly motivating.

Communicating and developing collaborative relationships with GPs was sometimes a problem, which some care homes had not yet managed to solve satisfactorily [16,18,20]. We did not have the resources to include the views of GPs and other relevant National Health Service staff in this study. However, a survey of GPs conducted as part of the evaluation of the GSFCH in Manchester found that the majority of GPs had a positive experience of end of life care in care homes [20]. They felt that the GSFCH increased staff confidence and found the out-of-hours handover form useful. There were concerns over continuity of care and the stability of the workforce, which may impact upon the sustainability of end of life care initiatives and confidence in care home staff. Seeing a sustained improvement in end of life care for residents is likely to increase GPs confidence in the programme. Direct enhanced service payments, thought to have enhanced the development of collaborative relationships between GPs and care homes implementing the GSFCH in Scotland, [18] might also help.

Some staff also described problems communicating with residents and their families. Although they felt that advanced care planning was important to foster choice for residents, there was some reluctance on the part of staff and residents to broach the subject of death, creating a barrier to both advance care planning discussions and offering bereavement support. Although this is not only a problem for staff from different cultures, cultural differences in attitudes towards death and dying do need to be considered. Lack of confidence in talking to residents and or their families about anticipatory planning in staff from different cultures was reported in the evaluation of the GSFCH in Scotland [18]. Studies conducted in Sweden [25] and the UK [26] have reported unwillingness of nursing home staff to talk about death. Reasons for this included: the belief that it was 'not healthy' for residents; personal difficulty in discussing death; and lack of time to have such conversations. Discussing death can also be an issue for some residents [16]. Good communication skills are necessary for staff to conduct such discussions, which cover a range of issues in addition to preferred place of death. There is a need to explore ways of facilitating such discussions, which takes cultural differences into consideration, and increased training is necessary to help staff understand cultural diversity and uncertain prognosis, improve communication skills and advance care planning, and change attitudes towards death and dying. However, education and training are necessary but not sufficient to improve end of life care in care homes. In view of the high staff turnover in many care homes, the way the care home functions needs to change to complement and encourage improved education and learning. It is also important to understand the factors resulting in residents' preferences not being met.

Staff attitudes towards death and dying can also be problematic when it comes to coding the predicted stage of illness and prognosis. This can be difficult to determine and some staff felt reluctant to "think in these terms". In her paper exploring the management of the dying process and the relationship between life and death in English nursing homes, Katherine Froggatt describes how care home staff are required to care for residents whatever their position on a continuum between living and dying, and the uncertainty inherent in the boundary between life and death for many residents [27]. She points out that in order to set dying residents apart from the living, knowledge is required that allows a resident to be defined as dying. However, many residents die following a process of gradual deterioration, occurring over months and years. She too, describes instances where residents have "bounced back".

We found that for some homes, the increased paperwork and lack of resources (time, money, and staff) were a problem. Some found it difficult to cope with the extra workload. In a study examining practitioners' perspectives on the GSF in primary care, the workload associated with the role of the GSF coordinator was one of the most common areas of concern [28]. Staffing, workload issues, and lack of financial resources are the reason some care homes dropped out of Phase II of the GSFCH [16], with staff shortages and time pressures being issues raised in the report on Phase I of the GSFCH [24]. Limited resources may result in some aspects of end of life care, such as after death care for staff and families, receiving less attention. The evaluation of Phase II of the GSFCH showed that only half the homes had a protocol for the bereaved and this did not increase by the end of the programme [17]. The extent to which staff, residents and families want bereavement support, and how best to provide it, needs to be explored.

### Strengths and weaknesses

The main strength of this work is that we have included a wide range of views and experiences. We have included the views of care home managers, nurses, care assistants, and residents and their families - in all care homes involved in the GSFCH in two areas of London. Three limitations of this research are: including homes in different phases of the GSFCH; not knowing how many GPs were implementing the GSF in their practices; and the generalisability of the findings to care homes without the external support provided to the homes in our study. Some homes were in the early stages of implementation of the GSFCH. Although it is useful to include the views of those in different stages of implementation, these were often based on beliefs and expectations rather than on experience. Knowing how many of the GP practices providing care to the homes in our study who were implementing the GSF would have facilitated interpretation of some of our findings. General Practitioners with experience of the GSF tasks and tools may be more supportive than those not involved in the programme. Finally, strong support from the local hospice and Care Home Support Team was available to all care homes in our study. Many care homes in England may not have access to this level of specialist palliative care support. The views and experiences of people working or living in homes implementing the GSFCH without such support may differ from those in our study.

### Future research

The residents and family members we interviewed had little knowledge of the GSFCH. They tended to talk about the care provided in very general terms, often focusing on recent or particularly memorable events, both good and bad. The possibility that the GSFCH does not necessarily result in better care was raised in the report of Phase II of the GSFCH [16]. It is, therefore, important to explore the impact of the GSFCH on outcomes for residents and their families using a validated measure. The UK End of Life Care Strategy has published recommendations for quality markers and measures for end of life care in care homes, which includes assessment of residents' views regarding the deaths of other residents [29]. There are inequalities in the capacity of care homes to implements end of life care tools such as the GSFCH and the LCP [10]. Only those homes that are judged to have a sufficiently developed organisation, clinical leadership and reasonably stable workforce are encouraged to implement such tools. Less fortunate homes may be excluded from the outside support resulting from implementing these tools. It is important to develop interventions to help care homes who do not reach the necessary standards to start or complete the GSFCH programme and care homes with personal care only (residential homes) to improve the end of life care of their residents.

## Conclusions

During the period the interviews were conducted, seven of the nine homes included in this study were progressing through various stages of implementing the GSFCH, and were generally positive about the programme. However, there were perceived barriers to be overcome to fully implement it. Some of these may be overcome as the GSFCH becomes more firmly embedded in these homes. Although this study focuses on a particular quality improvement programme, most of the barriers described by participants reflect the challenges that care homes face as private or third sector providers working with primary care services. Many of the participants' comments resonate with how care homes describe working with the National Health Service, as opposed to palliative care *per se*. Our findings demonstrate how difficult it can be to provide good care, even with frameworks in place and dedicated facilitation. There is a need to explore ways of overcoming these barriers and the impact of the different components of complex interventions to improve the care of residents. It is also important to investigate the impact of the GSFCH and other quality improvement programmes on a wider range of outcomes, particularly those focussing on the needs and experiences of residents and their families.

## Competing interests

The authors declare that they have no competing interests.

## Authors' contributions

SH designed the study, worked on the qualitative analysis and wrote the manuscript. CG and FS conducted the interviews, worked on the qualitative analysis and commented on drafts of the manuscript. IJH contributed to the design of the study and the commented on drafts of the manuscript. All authors have read and approved the final manuscript.

## Pre-publication history

The pre-publication history for this paper can be accessed here:

http://www.biomedcentral.com/1471-2318/11/31/prepub

## Supplementary Material

Additional file 1 **Topic guide**. Examples of questions and prompts from the topic guide. Click here for file

Additional file 2 **Framework approach**. Further details of the Framework approach to qualitative analysis, our treatment of the interview transcripts and the development of the thematic framework. Click here for file
