# Supplementary material for: Implementing a quality improvement programme in palliative care in care homes: a qualitative study
Source: BMC Geriatr. 2011 Jun 9;11:31. doi: 10.1186/1471-2318-11-31 (PMC3127758; doi:10.1186/1471-2318-11-31)
Supplement: Additional file 1 — Topic guide. Examples of questions and prompts from the topic guide. [file 1471-2318-11-31-S1.DOC]

**Additional File 1 Examples of questions and prompts from the topic guide**

| **Knowledge and perceptions of GSFCH**  Have you heard of the GSFCH?  Example of probes   - - What do they know about it?   - Perceived benefits for care homes, residents & their families?   - Perceived barriers?   **The 7 Cs**  *Communication*  The GSFCH involves maintaining a supportive care register to plan, record & monitor care of residents in the last 6-12 months of life. Have you used such a register in you care home?  Example of probes   - - If yes, did it help & how?   - Any issues regarding selecting residents reaching the end of life?   *Coordination*.  The GSFCH recommends a nominated coordinator for palliative care. Do you have such a coordinator in your care home?  Example of probes   - If yes, does it help & how? - Any issues regarding coordination?   *Control of symptoms*  The GSFCH provides forms & procedures for assessing, recording and acting upon physical and psychosocial symptoms. Have you used any of these?  Example of probes   - - If yes, did they help & how?   - Any issues identifying symptoms in residents?   *Continuity of care out of hours*  The GSFCH provides forms & procedures for maintaining continuity of care after hours. Have you used any of these?  Example of probes   - - If yes, did they help & how?   - Any issues regarding continuity and out of hours care?   *Continued learning*  The GSFCH recommends and offers training in end of life care for nurses and care assistants and forms & procedures for maintaining continuity of care after hours. Have you had any specific training in end of life care?  Example of probes   - If yes, did it help & how? - Any issues regarding training in end of life care in care homes?   *Carer Support*  The GSFCH recommends offering support for residents carers. This includes help with bereavement. Have you offered support to carers as part of the GSFCH?  Example of probes   - If yes, do you think they found it helpful? - Any issues regarding offering support for carers?   *Care of the dying*  The GSFCH recommends the use of the Liverpool Care Pathway (LCP) for residents in the last hours or days of life. Have you used the LCP in you care home?  Example of probes   - If yes, did it help & how? - If no, would they be interested in using the LCP? - Any issues regarding using the LCP? |
| --- |
