# Supplementary material for: Implementing a quality improvement programme in palliative care in care homes: a qualitative study
Source: BMC Geriatr. 2011 Jun 9;11:31. doi: 10.1186/1471-2318-11-31 (PMC3127758; doi:10.1186/1471-2318-11-31)
Supplement: Additional file 2 — Framework approach. Further details of the Framework approach to qualitative analysis, our treatment of the interview transcripts and the development of the thematic framework. [file 1471-2318-11-31-S2.DOC]

**Additional File 2 Further information on Framework Analysis, our treatment of the interview transcripts, and the development of the thematic framework**

The Framework method of analysis comprises five stages (1) Familiarisation (2) Identifying a thematic framework (3) Indexing (4) Charting (5) Mapping and interpretation.

1. Familiarisation

Three researchers (SH, CG and FS) immersed themselves in the raw data, by listening to the interviews, and reading the transcripts and field notes. They focused on all areas of the transcript related to the GSFCH, including spontaneously arising discussion about end of life care as well as in response to the specific probes about the GSFCH implementation.

2. Identifying a thematic framework

A thematic framework comprises a series of main themes and subtopics. Developing the framework for this study was an iterative process involving a succession of analyses, which involved modification of the framework based on the discrepancies between coders and then indexing (coding using the themes and sub-themes in the framework) using the latest version of the framework. The first framework drew on *a priori* issues: the perceived benefits of and barriers to aspects of the seven key tasks of the GSFCH (7 Cs), which were the main topic areas in the topic guide (i.e. the 7 Cs). CG and FS independently indexed 20 transcripts using this framework. SH, and FS met to discuss and agree the coding. The theme ‘communication’ was very complex and overlapped with other themes, therefore, sub-themes were agreed, which focused on some of the key GSFCH accreditation standards at that time. These themes, and sub-themes, became labels for the codes. Benefits and barriers were coded separately. Working within this framework the same 20 transcripts were independently re-coded by FS and CG. Following this, the authors met to agree the coding, with any disagreements resolved by discussion. This provided the basis for the rest of the analysis. At each stage, the text coded under each theme in the framework (using NVIVO) was printed, and the results of the discussions recorded on these ‘hard copies’.

3. Indexing

All data was indexed by FS using the new framework. All coding was then checked by CG & SH. The authors later met to discuss the coding, with any disagreements resolved by discussion.

4. Charting

Charts were created by FS for each of the themes identified. These contained participant’s quotes and allowed a visual aid to better organise the analysis and establish patterns within the data. There were separate charts for each theme and the data was grouped by stage of implementing the GSFCH (earlier or later), as the former homes had less experience of implementation.

5. Mapping and interpretation

The charts were used to describe the range of participants’ views in each theme and sub-theme, and the findings linked back to the literature. In view of the relatively straightforward nature of our aims, this stage was more descriptive than interpretative.
